# Can empathy be preserved in medical education?

**DOI:** 10.5116/ijme.5e83.31cf

**Published:** 2020-04-20

**Authors:** Astrid Seeberger, Annalena Lönn, Håkan Hult, Maria Weurlander, Annika Wernerson

**Affiliations:** 1 Division of Renal Medicine, Department of Clinical Science, Intervention and Technology (CLINTEC), Karolinska Insti-tutet, Stockholm, Sweden; 2 Department of Learning, School of Education and Communication in Engineering Science (ECE), KTH Royal Institute of Technology, Stockholm, Sweden

**Keywords:** Empathy, professionalisation, medical education, students´ experiences, reflection

## Abstract

**Objectives:**

The aim of this study was to investigate changes in empathy during medical education, as well as to identify promoters and inhibitors of empathy and analyse their roles.

**Methods:**

We used qualitative content analysis to examine 69 critically reflective essays written by medical students as a part of their final examination at the end of the medical program. The essays were based on previous self-evaluations performed each term and represented retrospective reflections on their professional development.

**Results:**

A majority of the students felt that their empathy did not decrease during medical education. On the contrary, many felt that their empathy had increased, especially the cognitive part of empathy, without loss of affective empathy. Many of them described a professionalisation process resulting in an ability to meet patients with preserved empathy but without being overwhelmed by emotions. They identified several factors that promoted the development of empathy: a multiplicity of patients, positive role models, and educational activities focusing on reflection and self-awareness. They also identified inhibitors of empathy: lack of professional competence and a stressful and empathy-hostile medical culture.

**Conclusions:**

Our analysis of these retrospective reflections by students suggests that empathy can be preserved during medical education, despite the presence of important inhibitors of empathy. This finding might be due to the presence of more potent promoters and/or to the fact that educational activities might result in a decreased susceptibility to empathy-decreasing circumstances.

## Introduction

During the last century, the shift of medicine toward science and science-based practices was followed by an empathy-aversion that has influenced decades of medical education into promoting a model of “detached care”.^1^ However, recent research has demonstrated the significance of empathy in clinical care. Empathic doctors have been shown to diagnose diseases more accurately than doctors lacking empathy.^2^ Furthermore, patients with empathic doctors are more prone to feel trust, participate in shared-decision making to a greater extent and show greater adherence to medical recommendations.^3^^-^^5^ Empathy is, therefore believed to have a significant influence on clinical outcome.^6^  In addition, empathy has been found to increase physicians´ job satisfaction whereas decreased empathy is associated with higher levels of burnout.^7^^-^^8^

Empathy is a complex competence that includes both affective and cognitive components.  Recent research has shown that emotions are important instruments of cognition and essential components of rational decision making.^9^^-^^10^ Consequently, affective and cognitive processes must cooperate in order to provide a complete understanding of the observed. However, there is still a belief in medical education that emotions are detrimental to good clinical judgment. Therefore, professional empathy is often defined as an intellectual competence that should be practised without getting emotionally involved with the patients. Unsurprisingly, a recent systematic review of the medical literature showed that 85% of articles defined professional empathy as a cognitive ability.^11^

Given the consensus that empathy is a desirable competence for doctors, the development of sustainable professional empathy is an important aim in medical education. Doctors who are continually confronted with suffering, fear and despair must be able to handle these emotionally challenging situations without losing the capacity to act in a highly-skilled, ethical and empathetic way. Three abilities are needed for empathy:  the cognitive capacity to assume the perspective of the observed, the ability to respond emotionally, and the ability to regulate one´s emotional response so that emotions do not hinder the cognitive response.

There is a prevailing view that empathy decreases during medical education. While several studies support this view,^12^^-^^13^, other studies are showing the opposite.^14^^-^^15^ Recent research has tried to identify factors that may promote or hinder the development of empathy.

According to Bourdieusian theories, medical education can be described as a process of adopting medical habitus.^16^ Medical students embody the values, attitudes and actions of the surrounding medical culture. A disjuncture between the formal curriculum that encourages empathy and the actual clinical practice (“the hidden curriculum”) that may discourage empathic behaviour has been shown to contribute to a decrease in empathy.^17^ Interestingly, the steepest decrease in self-reported empathy seems to occur when students start their clinical training.^18^

Other factors may also be important. In a German study based on interviews with 24 medical students either during their sixth term or during their final clinical year, empathic behaviour appeared to be influenced not only by the surrounding medical culture but also by aspects of the courses and by individual students and patient characteristics.^19^ However, the researchers did not investigate whether the empathy of the interviewed students changed during their medical education.

A review of studies on teaching empathy to medical students revealed the use of different interventional strategies aimed at promoting the development of empathy,^20^ but none of these studies investigated whether other promoters or inhibitors of empathy were present.

The aim of the present study was therefore to investigate the experiences of medical students regarding changes of empathy during medical education and to identify factors which the students believed had promoted or inhibited their development of empathy. Data were gathered from a qualitative self-report instrument: critically reflective essays written by the students at the end of their studies, based on standardised reflections performed each term.

## Methods

The research was conducted within an interpretative research tradition, using a qualitative research approach,^21^ to investigate medical students’ experiences of empathy and how they believed their empathy had changed. The data comprised critical reflection essays that the students wrote at the end of the medical curriculum, in which they reflected on their own development. The context of the study, the data collection and the analytical procedures are described in more detail below.

### Educational context

Medical education in Sweden takes 5.5 years. At the Karolinska Institutet (KI), each medical student is followed by a hospital-based physician serving as a mentor for the whole period of education. In the meetings with their mentors, the students reflect on their professional and personal development using structured self-evaluations according to a Canadian model modified for KI (CanMEDS, Canadian Medical Education Directives for Specialists).^22^

In addition, reflection seminars are held during terms five and six, allowing the students to reflect on their own and their patients´ thoughts, feelings and shortcomings using the Balint method.^23^ Common problems, such as lack of professional competence resulting in lack of self-confidence and impaired empathic abilities, are discussed in order to strengthen confidence and empathy. Students also learn strategies that help them to handle emotionally challenging situations and other forms of stress and to retain their empathy in medical cultures characterised by empathy-aversion. Research results are presented demonstrating that empathy is not only a feeling-good issue but an important tool to obtain optimal medical results.

At the end of their studies, the students write critically reflective essays as a part of their final examination. The essays, which represent retrospective reflections on the students´ own professional development, are based on the self-evaluations performed each term. The examiners assess the reflective capacity of the students in this part of the final examination, not the development per se or the results of the development. Reflective capacity is defined as a “critical analysis of knowledge and experience to achieve deeper meaning and understanding”, and the students are assessed using the criteria defined by Wald et al., which have been validated as a reliable way to assess medical students.^24^

### Participants and data collection methods

A convenience sample of 110 medical students was asked for permission to analyse the critically reflective essays written as a part of their final examination. The question, accompanied by a detailed description of the study was sent to the students after their essays had been assessed by the examiners, and the students had been informed of the results of the assessment. All of these students had passed the examination. A total of 69 students (36 women, 33 men) responded, and all agreed to our using their essays for research purposes. We followed ethical guidelines concerning research involving humans. The Regional Ethical Committee granted ethical approval. Students were informed both orally and in writing about the purpose of the study and their rights as participants. Only essays from students who chose to participate were collected. The essays were anonymised during the interpretation and analysis to protect the students’ integrity.

### Data analysis

We analysed 69 critically reflective essays. The first author performed the primary analysis; this was followed by an analysis by all members of our group.

We used a qualitative content analysis approach.^21^ After identifying the key points of the data using iterative comparative strategies that moved back and forth between data and analysis, we performed coding and categorisation by building groups of similar concepts while making systematic comparisons between observations and categories. We focused on the interpretational level, which meant that we went beyond the manifest content that was explicitly written and interpreted the latent content. The analysis was documented in notes and memos, and both the analyses and the resulting findings were discussed and agreed upon by all members of the research team to ensure that they were internally coherent and consistent. The essays were written and subsequently analysed in Swedish. The quotations presented in this article to illustrate the findings were translated into English.

## Results

### The development of empathy during medical education

A majority of the students reported that their empathy did not decrease during their medical education. On the contrary, many felt that their empathy had increased, especially the cognitive part of empathy, without a loss of affective empathy. Many also noted a qualitative change in their empathy: a professionalisation process that was not associated with a quantitative decrease in empathy. They had learned to keep affective and cognitive empathy in balance.

The students felt that they had learned to assume the perspective of the patients. A male student (no. 1) wrote:

“Previously I found it difficult to relate to depressed people´s thoughts, feelings and attitudes. Sometimes it struck me how I thought: ‘Pull yourself together; you just need to pick yourself up’. Due to experiences in my private life and in medical education, I became aware that this (the apathy of the patients) is a part of the disease process and that it is difficult for them to ‘just pick themselves up’. Now it is easier for me in meetings with depressed patients to have patience and be empathic in a way which is better for the patients and me. Now I know that it (depression) is a process which demands time, that no one wants to be in that situation, and that the kind of reception which I give them as a doctor can have a profound influence on the patients”.

This student was able to understand not only the patient but also the importance of his own role in the patient-physician relationship.

A female student (no. 2) described her development of professional empathy in the following way:

“At the beginning of your education/career, it is easy to take the patients problems home with you and be influenced by their sorrow so much that it becomes your own sorrow. That is something which I think you learn to let go by relating to your professional role. Of course, there will be particularly severe or traumatic experiences which will influence me as a human being, but I hope that I will improve in letting things go and not be absorbed too much. That does not mean that you lose your empathy, but that you find a new attitude towards challenging situations”.

The student had learned not to suppress her emotional response but to regulate it, which is an important part of the professionalization process. She was able to respond emotionally without being overwhelmed by emotions.

Only a minority of students felt that their empathy had decreased during medical education. Some of these students felt regret about the decrease because they were convinced of the value of empathy. They hoped that their empathy would increase when they felt greater self-confidence in their role as a doctor.

### Promoters and inhibitors of empathy

The development of empathy was described as a journey accompanied by events and factors in education and in private life that promoted or inhibited empathy.

### Education-associated promoters

#### Multiplicity of patients

Meeting many patients with different diseases, different backgrounds and different individual needs, preferences and values was considered an important promoter of professional empathy. As a female student (no. 3) wrote:

“When meeting patients with different personalities, you learn how differently people can feel and express their feelings. And you practice how to express your empathy in different ways”.

Meetings with patients both increased her ability to assume the patient´s perspective and developed her ability to express her empathy in a way that was adjusted to the individual patient.

#### Positive role models

We also found that positive role models promoted the cultivation of empathy. Another female student (no. 4) wrote:

“I found a role model, a senior consultant in obstetrics. Her warm personality combined with preciseness in medical judgement is something I aim for”.

This student chose a role model who demonstrated that clinical mastery needs to be accompanied by empathy in order to be a good doctor.

#### Reflection, self-awareness

Educational activities focusing on self-reflection and reflection together with teachers and inter-professional teams were reported to be important promoters of empathy. A male student (no. 5) wrote:

“Being aware of my own reactions and how they influence my meetings with the patients is a benefit both for the patients and me”.

Self-awareness enabled him to take care of the patients empathetically while minimising the emotional and cognitive toll for himself.

Another male student (no. 6) felt that his eyes were opened during a seminar in professional development concerning challenging meetings with patients and the traps you might fall into:

“We discussed an example showing how easy it is for an inexperienced intern to be affected by the patients and become captivated by this feeling and want to do more and perhaps promise too much. When this example was mentioned, I was struck by the fact that I was close to getting into this position last summer, when I met a young girl seeking asylum who had developed an apathetic syndrome, and her family, who were in a very vulnerable situation. I remembered that this family dominated my thoughts and that I easily could have done too much. The example opened my eyes, and I was surprised that I got into this position despite being convinced that I was aware of my feelings and attitudes”.

Reflections started by a seminar helped this student in his development of professional empathy, and he learned to regulate his feelings.

### Education-associated inhibitors

#### Lack of professional competence

Uncertainty regarding medical knowledge and medical skills was reported to diminish empathy. A male student (no. 7) wrote:

“I also remember that it is difficult to be empathetic when you do not understand the situation or the disease. I was at the rheumatology ward early in the early part of the course in clinical medicine and had to perform a bedside investigation of a girl of my age who had just received a diagnosis of SLE. She had only had one or two non-severe attacks of the disease, but she had many questions about long-term prognosis and fertility, and she started to cry in front of me. At this time, I did not know much about SLE, medication and the long-term effects of the disease and had never had concrete thoughts about pregnancy or about chronic disease in young people. I found it very difficult to understand her immediately and to act empathetically”.

This comment illustrates how difficult it is to assume the perspective of the patient and to respond empathetically without sufficient medical knowledge.

Being a competent professional includes having the ability to handle emotionally challenging situations without losing one´s affective and cognitive empathy. A female student (no. 8) who described herself as a genuinely empathetic person felt uncertain about her ability to take care of patients without becoming burned out during medical education. She, therefore, avoided emotional challenges, “for better and worse, in order to protect myself”, which resulted in a decrease in empathy. She had not learned to keep the balance between closeness and distance, which characterises professional empathy but had a tendency to identify with the suffering patients.

### A high-stress and empathy-hostile medical culture

The students also reported empathy-hostile clinical surroundings. A female student (no. 9) wrote:

“When I was patient-centred or empathetic, specific tutors told me that I worked too slowly and was too faint-hearted”.

She felt that some of her tutors disparaged her empathetic approach.

Students need a high degree of self-confidence and a strong belief in their image of a good doctor in order to resist a medical culture that is empathy-averse. A male student (no. 10) described the conflict between his own ideals and the clinical reality where doctors look at medical results on the computer screen but not at the patients. He felt split between two roles: *“the objective scientist and the medical humanist”.*

“When I´m feeling a little stiff in my back and my neck after hours at the desk, and I get up and go to a patient room in order to talk with some patients for a bit, the nurses glower at me… At this ward unit, doctors do not speak more than absolutely needed”.

Not all students have the self-confidence that this student showed. He continued to meet patients as individuals, not as diagnoses and was convinced that this approach was important for successful treatment.

Clinical work is often characterised by a high-stress load. A female student (no. 11) wrote:

“When I feel stressed, I lose my capacity to be empathetic”.

### Private experiences functioning as promoters or inhibitors

Private experiences were reported to play a role in not only the personal but also the professional development of the students. A male student (no. 12) wrote:

“During medical education, I met my partner. My girlfriend is wonderful in all respects although she, of course, has her deficiencies. We have different personalities, and she has a totally different background than I do, resulting in different values and experiences. Getting to know her deeply has given me better self-knowledge and greater insight into how differently people can think, feel, and act. This will help me to understand better the patients I meet. In addition, it will help me to cooperate with colleagues in the future”.

Giving birth to a child during medical education was found to promote empathy, illustrated by the following comment from a female student (no. 13).

“I feel empathy to a higher degree since I had children; in a way life, has become more important and more fragile for me”.

In contrast, another female student (no. 14) experienced a step back in the development of professional empathy when she gave birth to a child during the tenth term, demonstrating that the development of empathy is a highly individual journey.

“I have seen people die previously. Or get a cancer diagnosis. I felt sorrow, but I could handle it. I could keep work and life apart, patients feelings and my own. We were asked to define empathy, and as I understand the term, it includes the ability to understand other peoples feelings, and an ability to distinguish between their and your own feelings, so-called empathetic precision. Something happened to this third ability”.

This student (no. 14) gave a moving narrative returning from a home visit during the paediatrics course where she had met a dying child and the child´s family:

“Inside my door, my little whirlwind waits. My healthy child filled to the brim with life. I embrace him closely. And my tears come. I weep over the injustice of life, over the enormous sorrow behind the door we closed, over the barbaric cruelty that a child will die. I embrace and weep. Just now, professionally or not, I handle it in this way”.

As a mother herself, she identified with the mother of the dying child and lost her empathetic precision. She was no longer able to keep the balance between closeness and distance that characterises professional empathy. She concluded:

“I think that in many ways, my ability to act professionally in challenging situations was better during term nine than now. I have not grounded myself in a new situation. I have not found a functioning way to handle these situations. I hope that with more experiences, both as a clinician and as a mother, I will succeed”.

The narrative of this student illustrates that the development of empathy is not a straightforward process but can be characterised by ups and downs.

## Discussion

The main finding of the present study is that empathy can be preserved even in the presence of important inhibitors. This finding is in contrast to numerous previous studies showing a significant decrease in empathy during medical education, especially during the third year of medical education when the curriculum shifts toward patient-care activities.^12^^-^^13^^,^^18^ Circumstances causing this erosion of empathy are believed to include a lack of formal empathy training, inadequate mentoring, stress, and an empathy-hostile hidden curriculum.^25^ While these inhibitors were partially present in our study, they did not result in a decline of empathy.

Many other studies are in accordance with our finding that empathy did not decline during medical education.^14^^-^^15^ Several of these studies used a cross-sectional approach. For example, a large study investigating empathy in more than 1000 students in the United Kingdom and New Zealand found that students approaching the end of their undergraduate education did not report lower levels of empathy compared to those at the beginning of their course.^26^. However, this result does not prove that empathy can be preserved since the researchers did not follow one group of students during their medical education but measured empathy in two different groups.

Longitudinal studies demonstrating preservation or increase of empathy often use different interventional strategies, for example, communication skill training, role-playing, narrative writing, literature courses, reflective seminars and mindfulness training.^27^^-^^31^ However, in contrast to our study, these studies did not investigate whether inhibitors of empathy or other promoters were present.

In the present study, students reflected on factors that they believed to have promoted or inhibited their development of empathy. They identified three education-associated promoters: multiplicity of patients, positive role models and educational activities aiming to increase reflective abilities and self-awareness.

Clinical practice involving meeting a large number of patients with different diseases, different backgrounds and different individual needs, preferences and values was found to be helpful in the development of professional empathy. In this context, positive role models seem to play an important role, demonstrating that clinical practice can be performed in a skilled and empathic way, according to the aims of the formal curriculum. Our findings are in accordance with previous research showing that interactions with patients in medical practice and positive role models contribute to the cultivation of empathy.^32^^-^^33^

The students in the present study also felt that educational activities focusing on self-reflection, self-awareness and reflections together with teachers and with interprofessional teams were promoters of empathy. Previous research has found that critical reflection, especially self-reflection and reflection in dialectical or reflective discourse with others, represents a major element of transformative learning in professional development.^34^^,^^35^ Increased self-awareness has been shown to be an important component in developing empathetic clinicians.^36^ In addition, reflective seminars using the Balint method have been reported to increase the empathic abilities of medical students.^37^

The students also identified inhibitors of empathy: lack of professional competence and a medical culture characterised by stress and empathy-aversion. Insecurity or a lack of routine has previously been shown to decrease students´ empathy.^19^ This factor can be counteracted by an increase in medical knowledge and clinical skills, whereas the other inhibitor, a stressful and empathy-hostile medical culture, is more difficult to change.

International surveys have demonstrated that patients in Sweden experience that they are not treated as individuals with individual preferences, needs and values.^38^ In addition, clinical practice in Sweden is characterised by a high-stress load. This reality was evident in the students´ essays, which described high-stress and empathy-hostile clinical surroundings where patients were treated as diagnoses rather than as individuals:  findings which have previously been shown to decrease empathy.^25^^,^^32^

However, contrary to Bourdieusian theories proposing that medical students embody the values, attitudes and actions of the surrounding medical culture, our students felt that their empathic abilities were preserved and even increased during their studies despite the clinical surroundings that discouraged and obstructed empathic behaviour. This surprising finding has two possible explanations: the promoters of empathy might have been stronger than the inhibitors, or the students might have been less susceptible to empathy-inhibiting factors in medical culture.

It is necessary to consider the reliability of our results, which are based on qualitative analysis of retrospective critically reflective essays. Previous studies of empathy have often used quantitative methods such as the student version of Jefferson Scale of Physician Empathy (JSE-S) and/or the Davis Interpersonal Reactivity Index (IRI), which have the utility of being easily administered to a large number of students. However, it has been questioned whether these quantitative self-report instruments represent reliable measurements of clinical empathy.^39^  Studies have demonstrated that students´ scores on self-instruments show only a weak relation to observers´ scores of their behaviour, raising the question of whether self-report instruments measure the desirable view of the ideal doctor rather than true empathetic behaviour.^40^^,^^41^

We have two reasons for supposing that our approach elicits honest rather than expected responses, and hence that our data might capture a more accurate picture of the students' development. First, the students were trained in self-reflection. In self-evaluations performed each term according to a Canadian model (CanMEDS) modified for KI, the students became used to reflecting on their professional development without avoiding personal difficulties and problems. In addition, during the fifth and sixth terms, the students participated in seminars led by experienced clinicians, often psychiatrists, who encouraged them to reflect on their patients´ as well as their own feeling and thoughts, further developing their reflective abilities.

Second, the reflective essays used in our research are parts of the final examination. In order to pass, the students must be able to reflect in a critical analytical way, presenting descriptions of disorienting dilemmas, conflicts, challenges and issues of concern. The examiners assess the reflective capacity of the students, not the professional development per se or the results of the development (which are assessed in the other part of the final examination by other examiners). Consequently, students reporting that their empathy disappeared during their medical education will pass this part of the examination if they present a sufficient critical analysis of this development. This approach may have increased the students´openness and honesty. Moreover, it was clear that the students did not avoid reflections on failures and personal problems in their essays.

Our study does have some limitations. The present results are based on reflections by medical students in a Swedish context. In addition, we do not know whether these students actually practised empathy. Qualitative and quantitative studies are needed to investigate the relationship between subjective experiences and observed behaviour. We also do not know whether the empathy felt by the students at the end of their studies will last during their professional lives. We only have indications that we might have succeeded in fortifying them against lack of empathy in clinical practice. It remains for future research to show whether this fortification is sufficient.

## Conclusions

In summary, our study indicates that it is possible to preserve and even increase empathy during medical education despite the presence of important inhibitors such as a high-stress and empathy-averse medical culture that treats patients as objects rather than individuals (hidden curriculum). Our results lead us to the hypothesis that interventions in the formal curriculum aiming to cultivate empathy, such as reflective educational activities could be successful. Further research – both qualitative and quantitative – is needed to investigate this hypothesis.

### Acknowledgements

We thank the Swedish Research Council for financial support and all the students who permitted us to use their critical reflective essays for research purposes.

### Conflicts of Interest

The authors declare that they have no conflict of interest.
